# Comparative efficacy and dysmenorrhea score of 6 object-separated moxibustions for the treatment of Chinese patients with dysmenorrhea: A systematic review and network meta-analysis

**DOI:** 10.1097/MD.0000000000026185

**Published:** 2021-07-02

**Authors:** Zi-Xuan Wu, Min-Jie Cai, Pei-Dong Huang, Jia-Yun Chen, Zhao-Hui Lv, Xu-Yan Huang

**Affiliations:** a Guangzhou University of Chinese Medicine; b Guangdong Second Traditional Chinese Medicine Hospital, Guangzhou 510009, Guangdong; c Yunnan University of Chinese Medicine, Kunming 650500; d Guangdong Key Laboratory of Traditional Chinese Medicine Research and Development, Guangzhou 510095, Guangdong, China.

**Keywords:** dysmenorrhea score, network meta-analysis, object-separated moxibustions, primary dysmenorrhea

## Abstract

**Background:**

Primary dysmenorrhea (PD), one of the most common diseases in women, is known to be effective with object-separated moxibustion. However, because there is no large sample size for comparison, it is difficult to choose the best method for the clinical treatment of these different treatments. Therefore, our aim was to compare and rank different moxibustion methods to determine the most effective treatment method for PD.

**Materials and methods:**

A systematic search was carried out in PubMed, Cochrane Central Register of Controlled Trials, China National Knowledge Infrastructure, Wanfang Database, and Chinese Biomedical Literature, to identify the randomized controlled trials (RCTs) investigated the object-separated moxibustion is associated with dysmenorrhea, as well as we also manually checked the bibliographies of eligible studies and topic-related reviews, RCTs from their inception to May 1, 2020. Three investigators read the citations and excluded quasi-randomized trials and trials that were incomplete. We extracted data following a predefined hierarchy. We assessed the studies’ risk of bias in accordance with the Cochrane Handbook for Systematic Reviews of Interventions and certainty of evidence using the Grading of Recommendations Assessment, Development, and Evaluation (GRADE) framework. The primary outcomes were efficacy (response rate) and dysmenorrhea scores. We estimated the summary odds ratio (OR) and mean difference (MD) using pairwise and network meta-analyses with random effects. STATA software version 16.0, ADDIS software version 1.16.5, and R software version 3.6.1 were used to statistically analyze all data.

**Results:**

Fifty-six RCTs with 5550 patients were included, comparing 6 object-separated moxibustion therapies with acupuncture or oral medicine. All moxibustions were more effective than ibuprofen, with OR ranging between 6.75 (95%CI: 3.58 to 13.22) for moxibustion at the navel. For relieving pain which uses dysmenorrhea score to evaluate, mild moxibustion (MD = −1.42, −4.24 to 0.85) was more effective than others. A total of 24 (42.8%) of 56 trials were rated as having a high risk of bias, 31(55.4%) as moderate, and 1(1.8%) as low, and the certainty of the evidence was moderate.

**Conclusions:**

Mild moxibustion cannot only effectively treat PD but also relieve pain in comparison with ibuprofen. Although GRADE evidence indicate low to moderate for most comparisons, mild moxibustion seems to be an advisable option for PD treatment to relieve symptoms.

**Strengths and limitations of this study**

1. This study is the first Bayesian network meta-analysis to compare different object-separate moxibustions in the treatment of PD.
2. The quality of evidence will be assessed by the Grading of Recommendations Assessment, Development, and Evaluation system.
3. The publication bias of this study was analyzed using meta-regression.
4. Our research approach will focus on object-separated moxibustion methods, but without any discussion about the associated acupoint selection, the different quality of moxa sticks from different manufacturers, or analysis of the specific details of moxibustion techniques.
5. We will only retrieve data from Chinese and English databases, which could limit the available data or result in a language bias.

## 1 Introduction

In traditional Chinese medicine, primary dysmenorrhea (PD) is classified as “menstrual abdominal pain.” The main symptoms are periodic pain in the menstrual period, 1 week before and after menstruation, and radiation to the lumbosacral part. In severe cases, nausea, vomiting, cold hands and feet, sweating, and even fainting may occur.^[1]^ With the poor living habits of modern women, epidemiological investigations have found that the probability of PD among female college students is as high as 69.78%.^[2]^ Long-term PD can induce sadness, depression, and other negative emotions, seriously endangering the physical and mental health of women.^[3]^ Modern medicine believes that an imbalance in the secretion of prostaglandins (PGE2 and PGF2) is the main cause of PD.^[4]^ The main mechanisms of moxibustion are the heat effect, light effect, and the effect of moxa leaves and other drugs after combustion, while the regulation of endocrine, immune function, and neurological factors and the improvement of uterine microcirculation are the main mechanisms of moxibustion in the treatment of PD.^[5]^ Among them, the thermal effect is the main way for moxibustion to play a therapeutic role, and moxa smoke and moxibustion drugs also play an important role in the treatment.

The methods of traditional Chinese medicine (TCM) in the treatment of PD include acupuncture, massage, oral Chinese medicine, and physical instrument therapy. Among them, moxibustion therapy is widely used in clinics because of its good curative effect, simple operation, and less trauma. In the randomized controlled trials (RCTs) of moxibustion in the treatment of PD, their effectiveness and reduction of symptoms and signs have been reported. An increasing number of clinicians choose moxibustion as a treatment for PD, but at present, there is a lack of direct comparative studies of various moxibustion methods, and its relative effectiveness cannot be evaluated. It is difficult to choose the best treatment for PD and popularize it in clinical practice. Therefore, based on the method of network meta-analysis, this study compares and ranks the clinical effects of different object-separated moxibustion treatments on PD to provide more evidence of evidence-based medicine for clinicians to choose moxibustion for PD treatment to make a more reasonable choice of treatment methods.^[6]^

### 1.1 Data sources and searches

We followed the methods described by Wu et al.^[7]^ Several databases including PubMed, Embase, Central Register of Controlled Trials, Wanfang, China National Knowledge Infrastructure, and Chinese Biomedical Literature data were searched by 2 independent investigators to collect any RCTs related to moxibustion with PD. The data were searched from the inception by the beginning dates of the trials: May 14, 2020. We used a combination of medical subject heading and free word embedded in specific files involving title, keywords, and abstract: “Moxibustion,” “Primary dysmenorrheal” (Web Appendix 1 for full details about the search strategy). To guarantee the precision and recall ratio, we also checked the reference list of all relevant articles to identify additional studies.

### 1.2 Study selection

We pre-specified the inclusion criteria for our study: (1) the language and publication form are not limited, and it is a case–control study on the relationship between moxibustion and PD; (2) all patients were diagnosed with PD, and clear diagnostic criteria were required to be reported in this paper; (3) through reading the literature, we can calculate the odds ratio (OR) value and 95% confidence interval (CI) of the relationship between moxibustion and PD, or the estimated value and standard error of the logistic regression coefficient.

It was ineligible for the study in the following situations: (1) study on the non-standard name and unclear diagnosis of the disease; (2) only 1 report was published at the earliest, and the rest were excluded; (3) research lacked a control group; (4) repeated publications; and (5) report cases, experiments, and empirical studies.

The eligibility of studies for inclusion criteria was assessed independently by 3 reviewers (ZXW, MJC, and JYC) in triplicate. Any discrepancies were resolved by consensus between the 3 independent reviewers.

### 1.3 Data extraction and quality assessment

Data of expected outcomes of interest were extracted independently in triplicate from each included study by 3 investigators (ZXW, MJC, and JYC) using the pre-designed data extraction table, which included first author, publication year, sample size (male/female), and interventions (study group/control group). The risk of bias of the included studies was assessed according to the Cochrane risk of bias tool (ROB tool) 16. Because our observation indicators (efficiency) are subjective, although they have been presented in an objective form, there are still some deviations in the detection of the 3 results, so we conducted 3 deviation risk assessments. Additionally, the Grading of Recommendations Assessment, Development, and Evaluation (GRADE) framework were used to assess the quality of evidence for the primary outcomes contributing to each network estimate.

### 1.4 Data synthesis and analysis

#### 1.4.1 Methods for direct treatment comparisons

A standard pairwise meta-analysis was performed using the random-effects model. ORs with 95% CLs of efficiency were calculated as effect measures. The *I*^2^-statistic was calculated for heterogeneity as a measure of the proportion of the overall variation attributable to between-study heterogeneity.

#### 1.4.2 Methods for indirect and mixed comparisons

A random-effects network meta-analysis within a Bayesian framework was then performed. The OR for efficiency with 95%CI is summarized. We estimated the ranking probabilities for all treatments at each possible rank for each intervention. The treatment hierarchy was summarized and reported as the surface under the cumulative ranking curve (SUCRA) and mean ranks, which were considered secondary endpoints.

#### 1.4.3 Examination of assumptions in network meta-analysis (consistency and heterogeneity)

A loop-specific approach was used to evaluate the presence of local inconsistency in each closed loop. The node splitting method was used to assess the inconsistency of the model by separating evidence on a particular comparison between direct and indirect evidence. Global heterogeneity was assessed using the *I*^2^-statistic, and a predictive interval plot that incorporates the extent of heterogeneity was used to evaluate the extent of uncertainty in the estimated effect size locally.

#### 1.4.4 Publication bias and meta-regression

A comparison-adjusted funnel plot was used to detect potential publication bias in the results between small and large studies. All analyses were conducted using ADDIS 1.16.5 and STATA 16.0.

## 2 Results

### 2.1 Study characteristics

**Figure 1:**
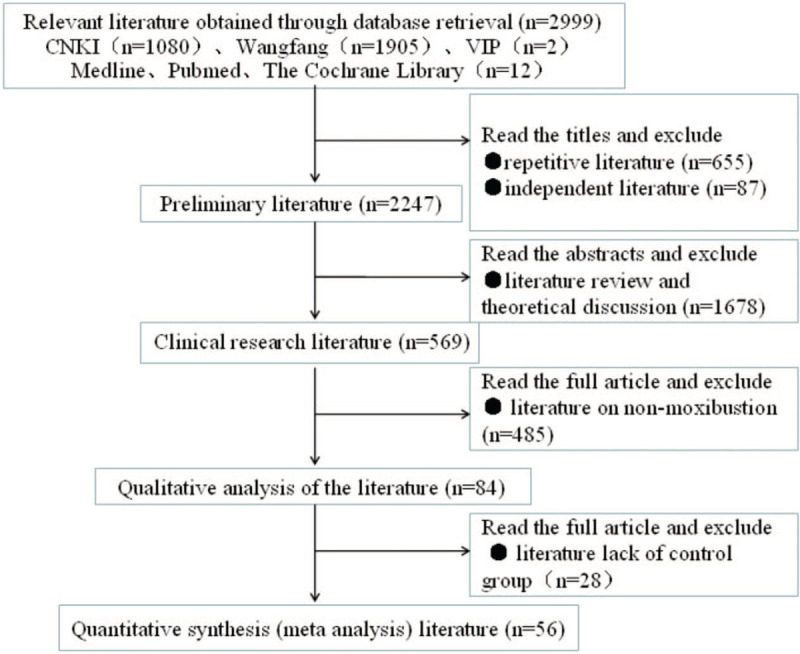
Flow chart of studies considered for inclusion.

**Figure 2:**
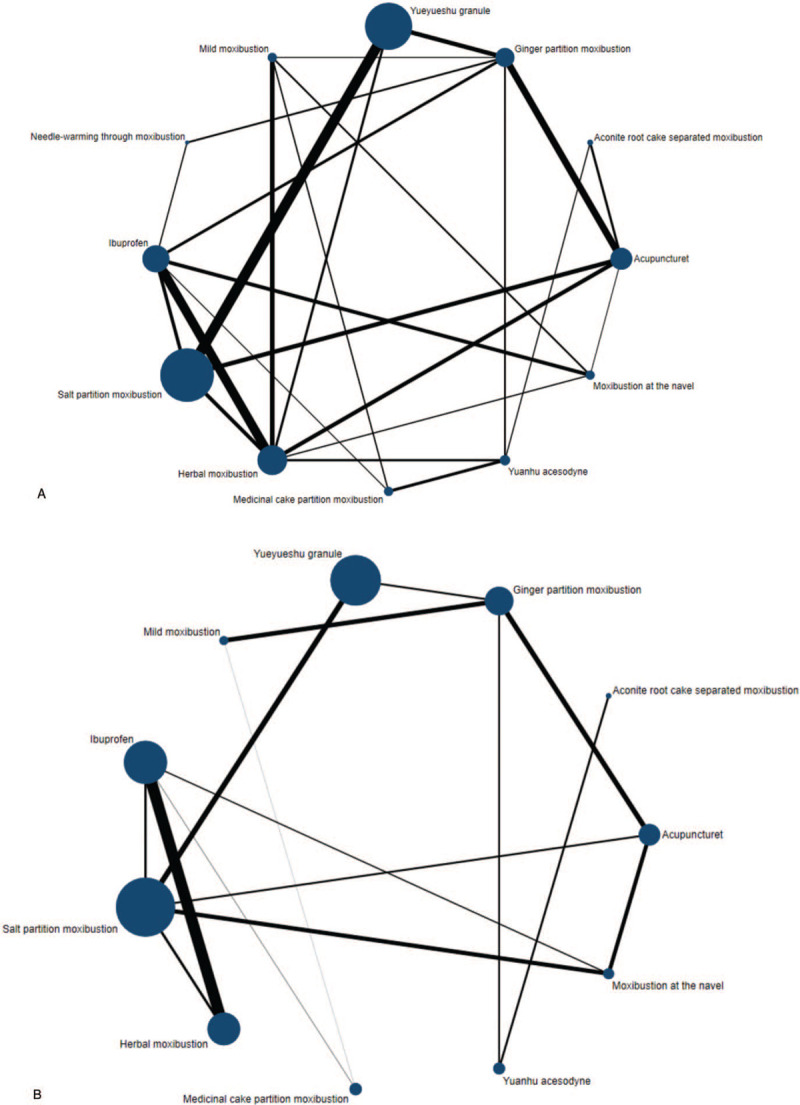
(A) Efficacy of eligible comparisons for network meta-analysis. (B) Dysmenorrhea score of eligible comparisons for network meta-analysis.

Overall, 56 trials met the inclusion criteria (Web Appendix 2 for the full reference list). A flow chart of the trial selection is shown in Figure 1. Twelve treatments were analyzed, including 7 different object-separated moxibustions, acupuncture, direct moxibustion, and 3 acesodynes. A total of 94.64% (53/56) of the trials were 2-arm studies and 5.36% (3/56) of trials were 3-arm studies (Appendix 3). Overall, 5500 patients contributed to the whole analysis, of which 5394 and 2175 patients contributed to 2 outcomes of efficacy and dysmenorrhea score (Fig. 2 for evidence network). Appendix 3 summarizes the characteristics of the included trials. The year of publication varied from 2001 to 2020. Of the 56 trials included, there were 12 items of acupuncture, 3 items of aconite root cake separated moxibustion, 11 items of ginger partition moxibustion, 14 items of Yueyueshu granule, 6 items of mild moxibustion, 1 item of needle-warming through moxibustion, 16 items of salt partition moxibustion, 16 items of herbal moxibustion, 4 items of medicinal cake partition moxibustion, 10 items of Yuanhu acesodyne, and 6 items of moxibustion at the navel.

### 2.2 Methodological quality and risk of bias results

In terms of the quality of included studies, allocation concealment was not clearly reported in 23.16% of the cases. In contrast, the blinding of participants and incomplete outcome data were appropriately described in the majority of studies (45.26% and 2.11%, respectively). A total of 22.11% of trials were open-label and 98.9% did not have selective reporting. Additionally, 24.21% did not report funding sources (Web Appendix 4 for risk of bias assessment).

The Jadad score was used to score the included studies. A total of 67.86 of the studies, 67.86% had 3 points, 19.6% had 4 points, and 12.5% had 2 points (Web Appendix 3 for Jadad score).

### 2.3 Results of a pairwise meta-analysis

**Figure 3:**
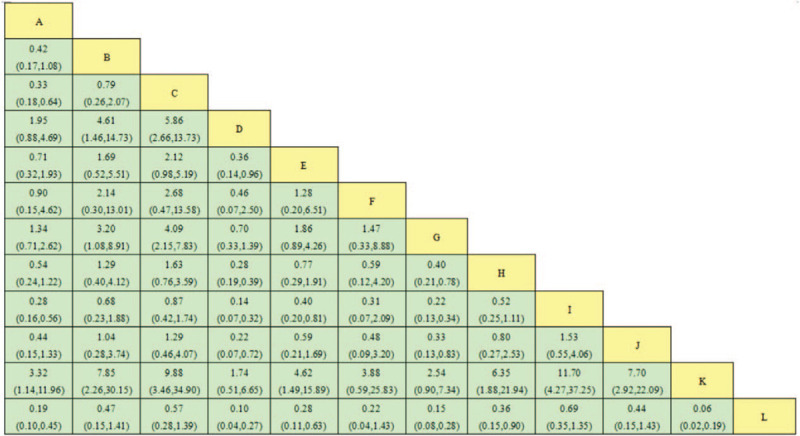
OR with 95%CI of network meta-analysis for efficacy. Note: 01: acupuncture; 02: aconite root cake separated moxibustion; 03: ginger partition moxibustion; 04: Yueyueshu granule; 05: mild moxibustion; 06: needle warming through moxibustion; 07: ibuprofen; 08: salt partition moxibustion; 09: herbal moxibustion; 10: medicinal cake partition moxibustion; 11: Yuanhu acesodyne; 12: moxibustion at the navel. CI = confidence interval, OR = odds ratio.

**Figure 4:**
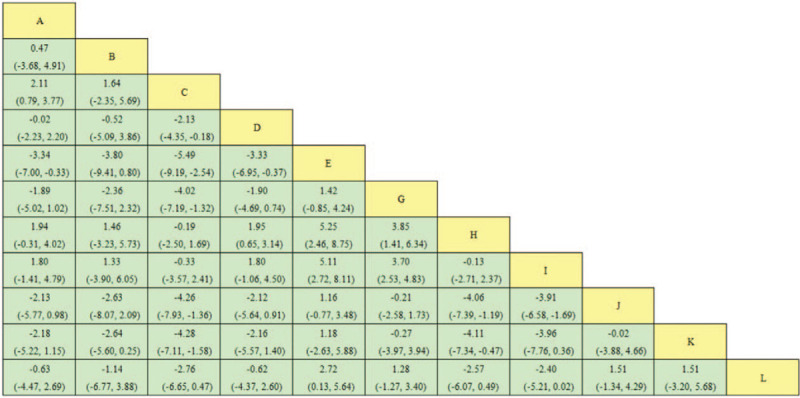
MD with 95%CI of network meta-analysis for dysmenorrhea score. Note: 01: acupuncture; 02: aconite root cake separated moxibustion; 03: ginger partition moxibustion; 04: Yueyueshu granule; 05: mild moxibustion; 06: needle warming through moxibustion; 07: ibuprofen; 08: salt partition moxibustion; 09: herbal moxibustion; 10: medicinal cake partition moxibustion; 11: Yuanhu acesodyne; 12: moxibustion at the navel. MD = mean difference, OR = odds ratio.

The effects of 6 object-separated moxibustions on efficacy and dysmenorrhea scores from the pairwise meta-analysis are shown in Figures 3 and 4. In terms of efficacy, all moxibustions were more effective than ibuprofen, moxibustion at the navel (OR, 6.75; 95%CI, 3.58–13.22) had the best curative effect. Mild moxibustion was more effective than ibuprofen (MD, −1.42; 95%CI, −4.24 to 0.85). All 6 types of object-separated moxibustion had no statistically significant reduction in pain compared with ibuprofen.

### 2.4 Results of network meta-analysis

The results of the network meta-analysis are reported in Figures 3 and 4. The efficacy of moxibustion at the navel was obviously better than ibuprofen (OR = 6.75, 95%CI: 3.58–13.22), followed by herbal moxibustion (OR = 4.62, 95%CI: 2.98–7.73), ginger partition moxibustion (OR = 4.09, 95%CI: 2.15–7.83), aconite root cake separated moxibustion (OR = 3.20, 95%CI: 1.08–8.91), medicinal cake partition moxibustion (OR = 3.02, 95%CI: 1.20–7.83), and salt partition moxibustion (OR = 2.48, 95%CI: 1.27–4.75). In terms of relieving pain, mild moxibustion was the best choice with MD (−1.42, −4.24 to 0.85), while the other 6 types of object-separated moxibustion were not statistically significant.

Above all, only mild moxibustion can not only effectively treat PD but also reduce pain in comparison with ibuprofen.

### 2.5 Inconsistency and heterogeneity

The baseline information for PD patients across treatment comparisons was relatively similar (Appendix 3). The test of global inconsistency did not detect any significant difference between the consistency and inconsistency models for any of the 2 outcomes. The test for local inconsistency showed that all loops were consistent for efficacy, and the dysmenorrhea score was consistent. Their 95% CI included 1 according to the inconsistency plots (Web Appendix 6 for assessment of inconsistency). The test of inconsistency from the node-splitting model showed no significant difference in most comparisons for all 2 outcomes; only 4 comparisons for efficacy and 3 comparisons for COX score showed significant differences between direct and indirect comparisons (Web Appendix 6 for assessment of inconsistency).

### 2.6 Meta-regression analyses

Meta-regression indicated that there is no small sample deviation (Web appendix 7).

### 2.7 SUCRA and ranking of all treatment

Web appendices 8 and 9 show the mean values of SUCRA for providing the hierarchy ranking of different treatments on efficacy and dysmenorrhea score. According to SUCRA, moxibustion at the navel ranked first on effectiveness, which is among all treatments with a probability of 69% and had a probability of 23% to rank second in the dysmenorrhea score for each corresponding outcome above. However, considering that not all trials were included (trials with active comparators such as efficiency and dysmenorrhea score were not included), the ranking might be highly biased and interpretation should be made with caution.

### 2.8 NMA result forest figure

Web appendix 10 is the forest plots for each moxibustion vs ibuprofen.

### 2.9 GRADE evaluation on the quality of evidence

According to GRADE, the quality of evidence ranged between very low and moderate but was rated as low and moderate for most comparisons (Web Appendix 5 for contribution summary of the risk of bias assessment and Web Appendix 11 for quality of evidence according to GRADE framework).

### 2.10 Patient and public involvement

No patients or members of the public were directly involved. Only data already existing in the literature and the aforementioned sources were used for this study.

## 3 Discussion

PD is a common gynecological disease that seriously affects daily life. Most scholars believe that its pathogenic factors are related to prostaglandins, estrogens, oxytocin, and other hormones in the body.^[8]^ In addition, the abnormality of calcium ions also has a certain correlation. Western medicine is mainly treated with non-steroidal anti-inflammatory drugs. In addition, oral contraceptives and calcium antagonists can also relieve symptoms to some extent. However, after drug withdrawal, there are a series of adverse drug reactions, such as endocrine disorders, irregular menstruation, and high recurrence rates. Moreover, there is no active role in the prevention and treatment of long-term complications.^[9]^ TCM syndrome differentiation and treatment have unique advantages in the treatment of “menstrual abdominal pain.” As a routine intervention measure, moxibustion has a certain curative effect in regulating endocrine hormones, improving immune function, regulating neurological factors, improving uterine microcirculation, and so on but its therapeutic mechanism is that which effective mechanism of moxibustion has not been clearly studied. Further studies are required.^[10]^

At present, the treatment methods of moxibustion are diverse, and there is also some evidence-based medicine to prove the effectiveness of all kinds of moxibustion in the treatment of PD. However, the curative effects of different methods are different, and clinical studies lack a comparison of the therapeutic effects of moxibustion and its related therapies; therefore, clinicians cannot clearly judge the differences in the therapeutic value of different moxibustion therapies in the clinical treatment of PD, which is not conducive to the promotion and application of moxibustion and its related therapies and the choice of the best treatment scheme. Therefore, we used network meta-analysis and systematic evaluation to compare and rank different moxibustion methods to solve the problem of lack of direct comparison in the original research, so as to provide reliable evidence-based medicine for the clinical application and curative effect judgment of different moxibustion methods in knee osteoarthritis.

In this study, by comparing and ranking the efficacy of different substance-separated moxibustions, it was found that only mild moxibustion was better than ibuprofen capsules. However, the first place in the evaluation of the curative effect is moxibustion at the navel. The anatomical position of the uterus is in the middle of the pelvis, generally between the bladder and rectum, but the filling degree or posture change of the bladder and rectum can change its position to a certain extent.^[11]^ The increase in PGF2 levels leads to vasospasm and contraction of blood vessels and smooth muscle in the uterus, and ischemia and hypoxia lead to dysmenorrhea.^[12]^*Artemisia argyi* leaves have antibacterial, anti-oxygen free radical effects, and enhanced immunity.^[13]^ By allowing drugs to be absorbed through the umbilical skin and combined with heat, object-separated moxibustion can absorb drugs into the human body through the skin and capillaries at a certain rate, thus giving full play to the role of warming meridians and dispelling cold and TCM.^[14]^ This is consistent with our results of umbilical moxibustion and herbal cake-separated moxibustion. Therefore, in the clinical treatment of dysmenorrhea, treatment should be prioritized in the umbilical region for mild moxibustion.

A major strength of our study is the comprehensive search and analysis of 6 object-separated moxibustion therapies compared with ibuprofen in a whole network with high quality. Furthermore, we conducted meta-regression by study characteristics to address the heterogeneity of the studies. Additionally, we assessed the quality of evidence and incorporated it into explaining the results using the GRADE framework. However, the different syndrome types of different research objects, different specific treatment methods, and different choice of moxibustion acupoints may cause deviations in the results to a certain extent, which limits the scope of this study. It is difficult to control the consistency of variables, different operation levels of the operator, the content of moxa leaf and other drugs in moxibustion, or the difference in treatment time and the deviation of course of treatment. Second, some comparisons were assessed as low quality in the GRADE framework, which might restrict the interpretation of the results. Finally, we did not have access to the original trial data, so we could not perform an individual patient data meta-analysis to properly assess the potentially relevant effect modifiers in our analyses.

## 4 Conclusions

In this study, through a literature review and reticular meta-analysis, it can be said that it has a certain reference value for clinicians to choose moxibustion as a TCM treatment in addition to the routine treatment of Western medicine in the treatment of PD, but the specific syndrome should also be combined with the actual situation of patients to make a reasonable choice of syndrome differentiation. There are some defects in the results of existing studies. Clinical workers are expected to report the results according to the CONSORT reporting standards in the future to provide a large number of high-quality RCT support to reduce the incidence of bias risk^[15]^; thus, the final evaluation results can provide a reliable evidence-based theoretical basis for guiding clinical drug selection.

## Author contributions

ZXW and MJC designed the study and drafted the manuscript. ZXW, MJC, and JYC extracted the data.

ZXW, MJC, and JYC evaluated RCT quality. ZXW, MJC, and JYC assessed the quality of the evidence using the GRADE framework. ZXW, MJC, and JYC verified the data, and ZXW and MJC analyzed the data. ZXW and MJC have revised the manuscript. ZXW, MJC, and JYC interpreted the results, incorporated comments for the co-authors, and finalized the manuscript. All the authors approved the final version of the manuscript.

**Methodology:** Zi-Xuan Wu, Min-Jie Cai, Pei-Dong Huang, Jia-Yun Chen, Zhao-Hui Lv.

**Writing – original draft:** Xuyan Huang.
